# Fecal Microbiota Transplant in Recurrent Clostridium Difficile Infections: A Systematic Review

**DOI:** 10.7759/cureus.24754

**Published:** 2022-05-05

**Authors:** Kunal Gupta, Mamatha Tappiti, Armaan M Nazir, Bhavya Koganti, Marrium S Memon, Muhammad Bin Aslam Zahid, Vignarth Shantha Kumar, Jihan A Mostafa

**Affiliations:** 1 Research, California Institute of Behavioral Neurosciences & Psychology, Fairfield, USA; 2 Neurosciences, California Institute of Behavioral Neurosciences & Psychology, Fairfield, USA; 3 Internal Medicine, California Institute of Behavioral Neurosciences & Psychology, Fairfield, USA; 4 Health Sciences, California Institute of Behavioral Neurosciences & Psychology, Fairfield, USA; 5 Psychiatry, California Institute of Behavioral Neurosciences & Psychology, Fairfield, USA

**Keywords:** pseudomembranous colitis, clostridium difficile colitis, clostridium difficile infection, stool transplant, fecal microbiota transplant

## Abstract

Fecal Microbiota Transplantation (FMT) is the process of transferring the fecal microbiome from a healthy donor to an individual with repeated multiple episodes of Clostridium difficile infection. It is also known as stool transplant. Fecal microbiota transplant is effective and safe in various studies, the approval from the Food and Drug Administration (FDA) remains pending. The main objective of this systemic review is to evaluate the efficacy and safety of stool transplant in studies with only treatment groups (FMT) and studies with treatment (FMT) and antibiotic (AB) groups and previous studies.

Online databases PubMed, PubMed Central, Science Direct, Google Scholar, and Embase were searched for relevant articles in the last five years (2016 to 2021) using automation tools. Following the removal of duplicates, screening of eligibility criteria, titles/abstracts, and quality appraisal were done by two authors independently.

In total, seven observational studies are in this review article. Out of the seven observational studies, five are retrospective and two prospective. Two of the five retrospective and one of two prospective studies have a control group. In both the prospective studies and one retrospective study, FMT efficacy of (68% to 93%) was demonstrated in the elderly population despite high index comorbidities. In the younger individuals with inflammatory bowel disease, and efficacy of 90% or above was found. The most common side effects were minor such as fever, abdominal pain, bloating, and flatulence. In one study, two cases of aspiration events occurred attributed to the gastroscopy route of donor feces delivery. There was no statistical significance in the incidence of diseases such as (allergies, autoimmune diseases, cancer, inflammatory bowel diseases, and neurological diseases like dementia and migraine).

Fecal microbiota transplantation has shown to be effective and safe in recurrent Clostridium difficile infections. Since very few pragmatic studies have demonstrated its efficacy and safety, their application is not well established. Robust studies, both observation and experiment, are required in the future to well-establish its effectiveness, safety in the treatment of recurrent Clostridium difficile infection.

## Introduction and background

Clostridium difficile infection (CDI) is one of the leading causes of healthcare-associated infections in the United States and possesses a significant burden on the health cost exceeding $4.8 billion [1]. Around 15,000 to 30,000 deaths are estimated to be associated with CDI [1]. Clostridium difficile infection is a disease caused by *Clostridium difficile*: a gram-positive, anaerobic bacteria spore-forming bacterium [2]. The use of broad-spectrum antibiotics leads to antibiotic-resistant pathogens and an increased risk of infections by lowering the protective microbiota community in the host [2]. 

Discontinuing the offending antibiotics and starting either metronidazole or vancomycin targeting the pathogen is the standard treatment [3]. However, the recurrence phenomenon of CDI is a common downfall despite successful treatment [3]. In patients who fail to respond to conventional therapy, the recurrence rate estimated is 18% to 35% after the first antibiotic use [2]. The estimated efficacy of antibiotic treatment for a first recurrence is 60%, a proportion that further declines in patients with multiple recurrences [3]. Therefore, there is a need for alternative treatments that can spare the host’s microbiota community.

Fecal microbiota transplant (FMT) is one such treatment that is effective in treating patients with recurrent and refractory clostridium infection. The benefit of fecal transplant is that it reduces the risk of multiple antibiotic resistance, and allergic reactions to antibiotics improve or even restore microbiota diversity in the gut [4]. The first role of FMT was described in 1958 by Eiseman et al. in four critically ill patients with refractory CDI using fecal enemas, and all had a complete clinical response [4].

Most studies have shown that FMT is effective in a cohort of Recurrent Clostridium Difficile Infection (RCDI) patients. However, the comparison between FMT and control (AB) groups in a cohort of RCDI was not performed. Therefore, in this review, the aim is to assess the efficacy and safety of FMT in RCDI patients and compare it with control groups.

## Review

Material and methods

Search Strategy

**Figure 1:**
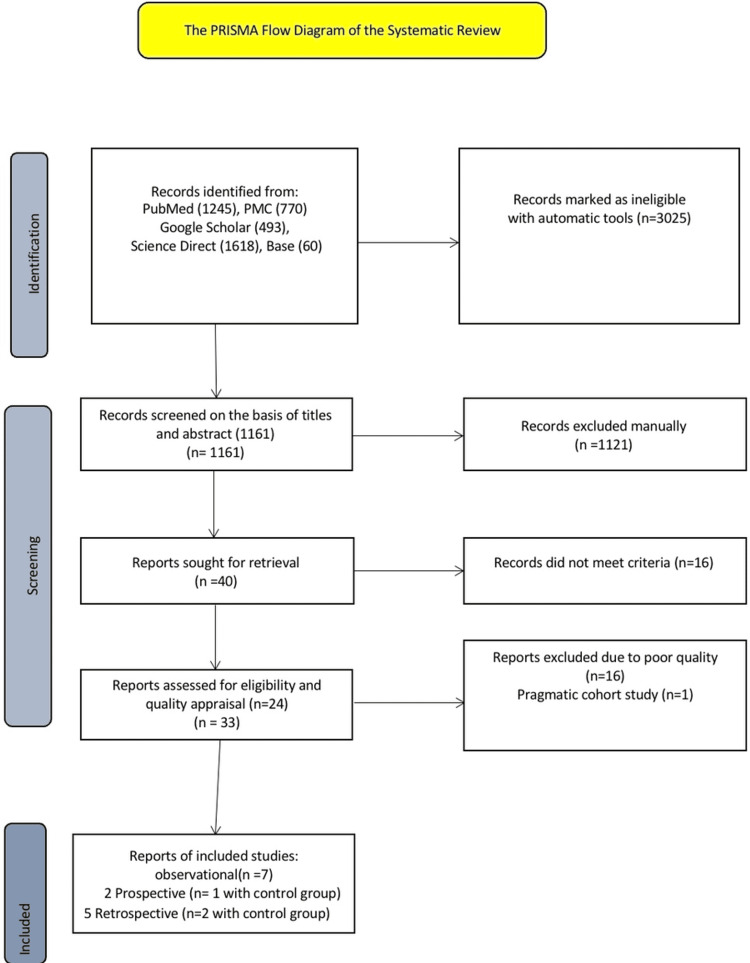
PRISMA flow chart (2020) guidelines PRISMA: Preferred Reporting Items for Systematic Reviews and Meta-Analyses A prospective study (n=1 with the control group) had matched baseline characteristics (age, sex, and no. of recurrences). A retrospective study (n=1 with the control group) had matched baseline characteristics (age, sex, and no. of recurrences).

The following databases: PubMed, PMC, Science Direct, Google Scholar, and Base databases in the last five years (2016 to 2021 ) were searched systematically using Medical Subject Heading (MeSH) terms and keywords: Fecal microbiota transplant, Fecal microbiome, Stool transplant, Clostridium difficile infection, Clostridium difficile colitis, Pseudomembranous colitis to collect the data. We performed a systematic search and applied filters: English, Humans, Age, Observations studies, Full text, Year of publication (2016 to 2021) on July 25, 2021, and came across 15 articles. PRISMA (Preferred Reporting Items for Systematic Reviews and Meta-Analyses) was followed for the article, as shown in Figure 1, which demonstrates the PRISMA flowchart followed for the article.

 Table 1 summarizes the search strategy for the review.

**Table 1:** Summary of the search strategy for the review MeSH: Medical subject heading Automatic filters: Age 18, human species, English language only, publication date (2016 to 2021), full text, and observational studies

Search strategy	Database	Total no. of articles	Total no. Of articles with automation tools	After removal of duplicates and screening	After full screening and quality appraisal
MeSH search: (Fecal microbiota transplant OR Fecal microbiome OR Stool transplant OR (Fecal microbiota transplant OR Fecal microbiome OR Stool transplant OR "Fecal Microbiota Transplantation/adverse effects"[Majr] OR "Fecal Microbiota Transplantation/methods"[Majr] OR "Fecal Microbiota Transplantation/therapeutic use"[Majr])) AND (Clostridium difficile infection OR Clostridium difficile colitis OR pseudomembranous colitis OR ("Clostridium Infections/complications"[Majr] OR "Clostridium Infections/diagnosis"[Majr] OR "Clostridium Infections/drug therapy"[Majr] OR "Clostridium Infections/etiology"[Majr] OR "Clostridium Infections/mortality"[Majr] OR "Clostridium Infections/physiopathology"[Majr] OR "Clostridium Infections/prevention and control"[Majr] OR "Clostridium Infections/therapy"[Majr]))	PUBMED	1245	15	9	2
Keywords: Fecal microbiota transplant, fecal microbiome, stool transplant, Clostridium difficile infection, Clostridium difficile colitis, pseudomembranous colitis	PUBMED CENTRAL (PMC)	770	458	8	3
Keywords: Fecal microbiota transplant, Clostridium difficile infection	SCIENCE DIRECT	1618	283	2	0
Keywords: Fecal microbiota transplant, Clostridium difficile infection	EMBASE	60	51	1	0
Keywords: Fecal microbiota transplant, fecal microbiome, stool transplant, Clostridium difficile infection, Clostridium difficile colitis, pseudomembranous colitis	GOOGLE SCHOLAR	493	354	4	2

Inclusion and Exclusion Criteria

Inclusion criteria: (a) study type: observational studies only; (b) language: English; (c) patients who were only 18 years or above; (d) recurrent Clostridium difficile infections; (e) species: humans.

Exclusion criteria: (a) animal studies; (b) gray literature; (c) articles other than English; (d) case reports, case series, systemic review, and metanalysis articles; (e) studies published before 2016; (f): cut-off score; below 50%.

The quality assessment tool used for observational studies was The Newcastle-Ottawa Scale.

Fecal microbiota transplant procedure (FMT)

Most studies used the lower gastrointestinal tract for delivery of donor feces, of which colonoscopy procedure followed by rectal route was most preferred. The amount of stool for each study was different. However, the type of stool was similar, either fresh or frozen or both. In addition, feces administered to the cohorts were from both related and not related donors in almost all studies.

Results

We obtained a total of 1161 articles after searching five online databases. All the duplicates then were removed, titles and abstracts, full texts screened, and a total of 40 articles were reviewed. After applying inclusion and exclusion criteria and quality appraisal using the Newcastle-Ottawa scale, a total of seven articles were eligible for this review. Two reviewers (MA and BK) had gone through the screening process, quality assessment, and data extraction.

Overall, the efficacy demonstrated was (68% to 100%) following repeat FMT. With a single FMT treatment (53% to 93%). The lowest efficacy rate (53%) was with FMT delivered by rectal and nasogastric tube route.

Discussion

Definitions

Clinical cure: It is defined as the resolution of diarrhea and, or positive/negative *C. difficile* toxin within 12 weeks or years. Overall cure: Clinical cure after one or repeat FMT. Safety: Occurrence of major and or minor events.

Elderly Population and Recurrent Clostridium Difficile Infection

Elderly patients are at more risk to get infected when compared to younger patients, likely attributed to lower immunity. Furthermore, using antibiotics to treat infections lowers the fecal microbiota, which is protective of the gut, and increases the risk of colonization of pathogens like *Clostridium difficile*. In addition, the risk factors such as advanced age, proton pump inhibitor use, gastrointestinal surgery, and presence of multiple comorbidities such as kidney disease, diabetes, etc.; further enhance the susceptibility to such pathogens resulting in diarrhea and colitis, impairing immune response and recurrence of infection [5]. In such patients, FMT is more promising and effective than targeted anti-CDI therapy.

In the study by Nowak et al., the overall success rate of FMT was 68% in cohorts of 47 RCDI with predominant three or more recurrent CDI episodes. Both rectal and nasogastric tube was used as the route of delivery of donor feces. There was no statistical significance in the Charlson Comorbidity Index in the cured and non-cured groups, but the Karnofsky Performance scale was high in patients with FMT success. Despite that, no association between the cure rate and the above-mentioned comorbidities. The efficacy seen was attained after more than one FMT in a few patients. It is unknown why some patients need more than one-time FMT treatment. Nevertheless, FMT has a high efficacy regardless of age and comorbidities. The study attributed the difference in cure rate with other studies to a small number of patients or the amount of donor feces material administered [6].

In another study by Girotra et al., in the elderly with major comorbidities: diabetes, hypertension, obesity, heart disease, vascular disease, neurological disease, chronic kidney disease, hyperlipidemia, hypothyroidism, antibiotics, etc., an efficacy of 93% was obtained. Both colonoscopy and enteroscopy were the routes of delivery of donor feces [7]. The difference seen in the effect may be due to a low number of patients. However, the cohorts followed up for a period of (25.4 +/- 12.8) was longer as compared to the study by Nowak et al., and no recurrences of R-CDI were documented over this period in the study by Girotra et al. This well supports that FMT is efficacious in elderly age patients despite the presence of comorbidities to cure RCDI. 

However, both the studies had no control groups to compare FMT efficacy to antibiotics (AB). In a randomized controlled trial by Van Nood et al., FMT was delivered via nasoduodenal tubes to RCDI patients when compared to a fourteen-day antibiotic course of vancomycin, with an efficacy rate of 94% vs. 31% [8]. In the clinical trial by Cammarota et al., FMT via colonoscopy showed an efficacy of 90% compared to 26% in the standard therapy group [9]. However, these trials were on a highly specific population. Van Nood et al. excluded hospitalized patients in the intensive care unit, and the average age of their patients was below 70. Cammarota et al. patients averaged 73 years with a Charlson score of 2. Hence, this population has been repeatedly underrepresented in previous studies [3].

Friedman et al. later conducted a study with a total of 34 patients well above 65 years of age, mean age of 80. A total of 39 patients (n=11) and AB (n =23) in FMT and AB groups were present in the study. Vancomycin was the antibiotic of choice in the antibiotic group. The route of donor feces delivery was colonoscopy and gastroscopy. Comorbidities measured by the Karnofsky Performance scale of the two groups were similar but small ( p=0.2 ). The Charlson Comorbidity Index was higher in both groups and statistically significant (p=0.043) [3]. The efficacy of FMT calculated was 90% and 39% in the antibiotic group (p=0.02), consistent with previous randomized clinical trials [3]. In this study, two significant adverse events of aspiration of fecal material occurred. In each, a gastroscopy was the route of delivery. In both patients, death occurred after around 10 days. One patient died of high intraabdominal pressure secondary to toxic megacolon despite surgical decompression. And the other, died of bilateral pneumonia, despite resuscitation attempts. Both the patients were above the age of 85 and had multiple comorbidities [3].

Although the mechanism of fecal microbiota transplantation is not yet well known, it is believed that restoration of intestinal flora composition is the reason behind cure with stool transplant [6]. Members of the phylum Firmicutes, including family Ruminococcaceae, are known butyrate producers, and an increase in the short-chain fatty acids (including butyrate) has been hypothesized as a possible mechanism. Post-FMT, an increase in the ratio of Phylum Firmicutes/Proteobacteria may be linked to increased butyrate productions leading to resolution of RCDI [7]. In addition, FMT is effective in RCDI patients with inflammatory bowel diseases, etc., which further supports the use of this procedure as a treatment [10,11]. A recent study on the long-term effects on intestinal mucosal and microbiota following fecal transplant has shown that FMT induces a profound effect on microbiota changes, therefore explaining the high efficacy of FMT in RCDI [12].

Inflammatory Bowel Disease (IBD) and Recurrent Clostridium Difficile Infection

Like the elderly, there is a high risk of association with RCDI in patients with inflammatory bowel disease. IBD is a risk factor for CDI itself. Several factors have an association between the two: immunosuppressive medications and antibiotic therapy that induces dysbiosis - an alteration in microbiota diversity that increases colonization of Clostridium, and lower immune and nutritional status [13]. A recidivism study showed that in comparison to the general population, IBD patients are 33% prone to RCDI [14]. Also, the recidivism study showed a 20 times increase in the risk of colectomy in inflammatory bowel disease patients with one single episode of Clostridium difficile infection [14]. All these factors led to the increased importance of FMT in RCDI patients with IBD as comorbidity, although few studies have shown that FMT in IBD patients is less effective [15]. 

However, FMT is effective in patients with IBD in treating RCDI, although there is not much improvement in IBD outcomes [10,11]. In addition, the efficacy in the IBD is similar to the non-IBD elder population [10].

One study in the IBD population, over three months follow-up in a cohort of 54 RCDI patients demonstrated an efficacy of 79%. With repeat FMT the efficacy reached a level of 90% [11]. Another study showed an efficacy of 71.4%, which reached a level of 90.4% with repeat FMT over a follow-up period of one or more years [10]. The results in these two studies are consistent. In both these studies, patients were of similar age groups, and both groups were on common medications like aspirin (ASA), steroids, immunomodulators, and biologics (anti-TNF) alone or in combination at the time of FMT. Likewise, in the non-IBD patients above the age of 60, the level of efficacy was 84.2% [10]. In the seven studies included in this review, FMT is effective and safe for IBD and the elderly population. 

Most studies in this review suggest FMT is effective in patients with multiple recurrences with an efficacy range ( 68% to 100% ). Moreover, in RCDI groups with three or more recurrences, FMT is clearly beneficial. This benefit of FMT has been well established in a cohort study consisting of 113 RCDI patients, of which 77 patients with three or more recurrences were in the FMT (n=52) and non-FMT (n=25) groups. The remaining (n=36) patients with less than three recurrences were included in the non-FMT group. The baseline characteristics and comorbidities were comparable in both groups except for pulmonary disorders. The non-FMT group (n=25) met the criteria for FMT procedure but were included in the standard (AB), citing patients' preference and or safety concerns from comorbidities. Over a follow-up period of three months follow-up, in the FMT and non-FMT groups with three or more recurrent episodes, the recurrence rate was 4.5% and 16.7%, respectively. However, in the non-FMT group with less than three recurrences, the recurrence rate was 8.8%. When the comparison is made between non-FMT groups with three or more and less than three recurrent episodes when compared, the rate was two times higher in the non-FMT group with three or more recurrent episodes [16]. The study also reported an overall improvement in health, energy level, appetite, and weight in the FMT group compared to the non-FMT group.

In the six studies included in this review, there were minor events such as abdominal pain, flatulence, bloating, and fever. There were major events but were not attributable to FMT treatment in the IBD populations in two studies included in this review. These events were: ileal resection, colectomy, hospitalization for IBD flare, small bowel obstruction, cytomegalovirus infection, and pancreatitis. It shows that FMT is safe in RCDI patients with IBD. However, the safety over a long period was not known. Also, there remain concerns regarding the transmission of infections or diseases to FMT recipients through donor feces microbiome. A systematic review of 536 patients receiving FMT treatment showed no risk of transmission of diseases to FMT recipients from donor feces. As the follow-up period was less than a year, the results obtained cannot be estimated for long-term effects. In addition, many case reports have mentioned the side effects like fever, abdominal pain, diarrhea, exhaustion, flatulence, fatigue, diverticulitis, bacteremia, norovirus gastroenteritis, cytomegalovirus infection, and activation of ulcerative colitis over a short period [17-23]. In addition, a high prevalence of functional gastrointestinal disorders has been noted in patients with Clostridium difficile infection. It has also been hypothesized that the use of antibiotics causes inflammatory bowel syndrome-like symptoms linked to CDI treatment [24].

Table 2 summarizes the outcomes of recurrent Clostridium difficile infection following fecal microbiota transplant.

**Table 2:** Summarizes the outcomes of recurrent Clostridium difficile infection following fecal microbiota transplant * Not recorded FMT: fecal microbiota transplant, AB: antibiotic, IBD: inflammatory bowel disease. Non-IBD: non-inflammatory bowel disease

STUDY	CLINICAL CURE	OVERALL CURE	RECURRENCE	SAFETY/ADVERSEEVENTS (MINOR/ MAJOR)
Friedman et al., [3]	Resolution of diarrhea by day three & negative C. difficile in stool within 10 wks. without relapse	82% FMT 39% AB after single FMT	2 in FMT 14 in AB	0/2
Nowak et al., [6]	Resolution of diarrhea & positive C. difficile toxin after 10 weeks	68% with repeat FMT	14	8/0
Girotra et al., [7]	Resolution of diarrhea, abdominal pain & negative C. difficile toxin within 4 to 12wks	93% after single FMT	0	5/0
Tabbaa et al., [10]	Diarrhea free & C. negative C. difficile toxin more than a year	90.4% IBD 84.2% non-IBD with repeat FMT	6 in IBD 12 in non-IBD	*/6 in IBD
Fischer et al., [11]	Resolution of diarrhea by day three & negative C. difficile in stool within 10 wks. without relapse Resolution of diarrhea &/OR negative C. difficile toxin within 12 wks. of FMT without the need of anti-CDI therapy.	90%	7	*/8
Shin et al., [16]	*	*	2(4.5%) in FMT 4(16.7%) in AB	*
Jalanka et al., [25]	Resolution of symptoms OR C. difficile culture or toxin negative over 3.8 yrs.	100% with repeat FMT * AB	0	*/28 FMT=11 AB=17

Table 3 summarizes the characteristics of the studies included. We included a total of seven studies. All were cohort studies, either retrospective or prospective.

**Table 3:** Summarizes the characteristics of the studies included in this review — Not recorded. FMT: fecal microbiota transplant. AB: antibiotic. RCDI: recurrent Clostridium difficile infection. REF.CDI: refractory Clostridium difficile infection. IBD: inflammatory bowel disease. Non-IBD: non-inflammatory bowel disease.

STUDY	DESIGN	AGE	SEX (F)	INDICATION	NO. OF RECURRENT EPISODES	FMT GROUP	CONTROL (AB) GROUP
Friedman et al., [3]	Prospective	82 (60-94)	21	RCDI	≥1	11	23
Nowak et al., [6]	Retrospective	70	33	RCDI	1-2 (n=13) ≥3(n=34)	47	—
Girotra et al., [7]	Prospective	80 ± 6.49 (70-91)	23	RCDI despite AB treatment	≥3	29	—
Tabbaa et al., [10]	Retrospective	57.5±20.2	54	RCDI/REF. CDI	>1	78 IBD (n=21) NON-IBD (n=57)	
Fischer et al., [11]	Retrospective	45.42±17.3	39	RCDI	>3	54	—
Shin et al., [16]	Retrospective	64 (20-92)	80	RCDI	≥3(n=77) <3 (n=36)	52	61
Jalanka et al., [25]	Retrospective	56 (22-91)	84	RCDI	≥3	45	39

Table 4 summarizes the characteristics of fecal microbiota transplant procedures in recurrent clostridium infections.

**Table 4:** Summarizes the characteristics of the FMT procedures in RCDI patients — Not recorded FMT: fecal microbiota transplant. RCDI: recurrent Clostridium difficile infection. PEG: percutaneous gastrostomy tube. EGD: upper gastrointestinal endoscopy. G/J: gastrostomy and/or jejunostomy

STUDY	ROUTE	DONOR RELATED NON-RELATED	STOOL TYPE	AMOUNT	FMT & AB
Friedman et al., [3]	Colonoscopy (32) Gastroscopy (2)	Both	Fresh	—	NO
Nowak et al., [6]	Rectal (42) Nasogastric tube (5)	Both	Fresh (33) Frozen (14)	30g/cc	NO
Girotra et al., [7]	Colonoscopy& Enteroscopy	Both	—	270cc (colonoscopy) 180cc (enteroscopy)	NO
Tabbaa et al., [10]	Colonoscopy (62) Sigmoidoscopy (3) G/J tube (2) Enema (1) Enteroscopy (1)	Both	—	250-1200ml	NO
Fischer et al., [11]	Colonoscopy	Both	Fresh	—	NO
Shin et al., [16]	Colonoscopy (50) EGD (1) PEG (1)	—	Frozen	—	NO
Jalanka et al., [25]	Colonoscopy	Both	Fresh, Frozen	—	NO

To address these long-term implications of FMT and compare its efficacy over a long period, Jalanka et al. conducted a retrospective observational study on 84 participants: FMT ( n=45 ) and AB ( n=39 ) over three years [25].

The study showed statistically no significant differences in the incidence of allergic diseases and prevalence of diseases like IBD, cancer, autoimmune diseases, and neurological diseases (migraine and dementia), rather there was a statistically significant improvement in bowel functions between the groups, respectively (p=.016). With regards to inflammatory bowel syndrome (IBS) like symptoms, a borderline statistical significance (p=0.06) was seen [25]. The study also reported mental health improvement in the FMT group compared to the AB group, reaching a borderline statistical significance (p=0.06). This well suggests that fecal microbiota transplant is safe for treatment in patients with recurrent Clostridium difficile infection and beneficial over antibiotic treatment in the long term. 

Limitations

Most of the cohort studies mentioned in this review were retrospective in nature. Hence, the risk of recall bias is well understood. The quality appraisal of the studies in this review was only 50%. Three observational studies without a control group were present. Some observational studies with cohorts of refractory and recurrent *Clostridium difficile* were included. Hence, this may have overestimated the effect of FMT. Data on the weight of stool of donors in four studies and the type of feces delivered in two studies to RCDI cohorts were not reported. Most of the studies mentioned that patients were receiving antibiotics that were discontinued days to weeks before the FMT procedure. Hence, this made it difficult to estimate the positive effect of FMT alone. Also, why in some studies, a higher cure rate was demonstrated after repeat FMT is not described. This review entails the efficacy and safety of FMT in the elderly and IBD patients. However, whether FMT improves inflammatory bowel disease in RCDI cohorts is not mentioned.

## Conclusions

In summary, fecal microbiota transplant is effective and safe in recurrent clostridium infection in elderly and inflammatory bowel disease populations. It is an alternative to treating Clostridium difficile infection in patients with multiple recurrences and those who are unresponsive or less responsive to standard antibiotic therapy. Although the colonoscopy route was the most common route of donor feces delivery in most studies, randomized control trials are needed to compare this effect of fecal microbiota transplantation over another method of delivery. Likewise, clinical trials of large sample sizes are necessary to compare FMT efficacy and conventional antibiotics. In addition, clinical trials to determine the effect of fecal microbiota transplantation on inflammatory bowel diseases with RCDI and its outcomes vs. the AB group in the future.

## References

[1] Fu Y, Luo Y, Grinspan AM (2021). Epidemiology of community-acquired and recurrent Clostridioides difficile infection. Therap Adv Gastroenterol.

[2] Gough E, Shaikh H, Manges AR (2011). Systematic review of intestinal microbiota transplantation (fecal bacteriotherapy) for recurrent Clostridium difficile infection. Clin Infect Dis.

[3] Friedman-Korn T, Livovsky DM, Maharshak N (2018). Fecal transplantation for treatment of Clostridium difficile infection in elderly and debilitated patients. Dig Dis Sci.

[4] Aas J, Gessert CE, Bakken JS (2003). Recurrent Clostridium difficile colitis: case series involving 18 patients treated with donor stool administered via a nasogastric tube. Clin Infect Dis.

[5] Leffler DA, Lamont JT (2015). Clostridium difficile infection. N Engl J Med.

[6] Nowak A, Hedenstierna M, Ursing J, Lidman C, Nowak P (2019). Efficacy of routine fecal microbiota transplantation for treatment of recurrent Clostridium difficile infection: a retrospective cohort study. Int J Microbiol.

[7] Girotra M, Garg S, Anand R, Song Y, Dutta SK (2016). Fecal microbiota transplantation for recurrent Clostridium difficile infection in the elderly: long-term outcomes and microbiota changes. Dig Dis Sci.

[8] van Nood E, Vrieze A, Nieuwdorp M (2013). Duodenal infusion of donor feces for recurrent Clostridium difficile. N Engl J Med.

[9] Cammarota G, Masucci L, Ianiro G (2015). Randomised clinical trial: faecal microbiota transplantation by colonoscopy vs. vancomycin for the treatment of recurrent Clostridium difficile infection. Aliment Pharmacol Ther.

[10] Tabbaa OM, Aboelsoud MM, Mattar MC (2018). Long-term safety and efficacy of fecal microbiota transplantation in the treatment of Clostridium difficile infection in patients with and without inflammatory bowel disease: a tertiary care center's experience. Gastroenterology Res.

[11] Fischer M, Kao D, Kelly C (2016). Fecal microbiota transplantation is safe and efficacious for recurrent or refractory Clostridium difficile infection in patients with inflammatory bowel disease. Inflamm Bowel Dis.

[12] Jalanka J, Mattila E, Jouhten H, Hartman J, de Vos WM, Arkkila P, Satokari R (2016). Long-term effects on luminal and mucosal microbiota and commonly acquired taxa in faecal microbiota transplantation for recurrent Clostridium difficile infection. BMC Med.

[13] Freeman HJ (2008). Recent developments on the role of Clostridium difficile in inflammatory bowel disease. World J Gastroenterol.

[14] Razik R, Rumman A, Bahreini Z, McGeer A, Nguyen GC (2016). Recurrence of Clostridium difficile infection in patients with inflammatory bowel disease: the RECIDIVISM study. Am J Gastroenterol.

[15] Khoruts A, Rank KM, Newman KM, Viskocil K, Vaughn BP, Hamilton MJ, Sadowsky MJ (2016). Inflammatory bowel disease affects the outcome of fecal microbiota transplantation for recurrent Clostridium difficile infection. Clin Gastroenterol Hepatol.

[16] Shin JH, Chaplin AS, Hays RA (2019). Outcomes of a multidisciplinary clinic in evaluating recurrent Clostridioides difficile infection patients for fecal microbiota transplant: a retrospective cohort analysis. J Clin Med.

[17] Mandalia A, Kraft CS, Dhere T (2014). Diverticulitis after fecal microbiota transplant for C. difficile infection. Am J Gastroenterol.

[18] Quera R, Espinoza R, Estay C, Rivera D (2014). Bacteremia as an adverse event of fecal microbiota transplantation in a patient with Crohn's disease and recurrent Clostridium difficile infection. J Crohns Colitis.

[19] De Leon LM, Watson JB, Kelly CR (2013). Transient flare of ulcerative colitis after fecal microbiota transplantation for recurrent Clostridium difficile infection. Clin Gastroenterol Hepatol.

[20] Satokari R, Fuentes S, Mattila E, Jalanka J, de Vos WM, Arkkila P (2014). Fecal transplantation treatment of antibiotic-induced, noninfectious colitis and long-term microbiota follow-up. Case Rep Med.

[21] Schwartz M, Gluck M, Koon S (2013). Norovirus gastroenteritis after fecal microbiota transplantation for treatment of Clostridium difficile infection despite asymptomatic donors and lack of sick contacts. Am J Gastroenterol.

[22] Kunde S, Pham A, Bonczyk S (2013). Safety, tolerability, and clinical response after fecal transplantation in children and young adults with ulcerative colitis. J Pediatr Gastroenterol Nutr.

[23] Hohmann EL, Ananthakrishnan AN, Deshpande V (2014). Case 25-2014 — a 37-year-old man with ulcerative colitis and bloody diarrhea. N Engl J Med.

[24] Villarreal AA, Aberger FJ, Benrud R, Gundrum JD (2012). Use of broad-spectrum antibiotics and the development of irritable bowel syndrome. WMJ.

[25] Jalanka J, Hillamaa A, Satokari R, Mattila E, Anttila VJ, Arkkila P (2018). The long-term effects of faecal microbiota transplantation for gastrointestinal symptoms and general health in patients with recurrent Clostridium difficile infection. Aliment Pharmacol Ther.

